# Relapsed diffuse large B-cell lymphoma present different genomic profiles between early and late relapses

**DOI:** 10.18632/oncotarget.9793

**Published:** 2016-06-02

**Authors:** Julien Broséus, Gaili Chen, Sébastien Hergalant, Gérard Ramstein, Nicolas Mounier, Jean-Louis Guéant, Pierre Feugier, Christian Gisselbrecht, Catherine Thieblemont, Rémi Houlgatte

**Affiliations:** ^1^ Inserm U954, Faculty of Medicine, Nancy, France; ^2^ Hematology, Laboratory Department, University Hospital of Nancy, Nancy, France; ^3^ ZhongNan Hospital of Wuhan University, Wuhan, China; ^4^ LINA DUKe, UMR 6241, Université de Nantes, Nantes, France; ^5^ Hemato-oncology, University Hospital of l’Archet, Nice, France; ^6^ Biochemistry, Laboratory Department, University Hospital of Nancy, Nancy, France; ^7^ Hematology Department, University Hospital of Nancy, Nancy, France; ^8^ APHP, Saint-Louis Hospital, Hemato-Oncology Department, Paris, France; ^9^ Paris Diderot University-Sorbonne Paris-Cité, Paris, France; ^10^ DRCI, University Hospital of Nancy, Nancy, France

**Keywords:** diffuse large B-cell lymphoma, early relapse, late relapse, genomics, copy number variations

## Abstract

Despite major advances in first-line treatment, a significant proportion of patients with diffuse large B-cell lymphoma (DLBCL) will experience treatment failure. Prognosis is particularly poor for relapses occurring less than one year after the end of first-line treatment (early relapses/ER) compared to those occurring more than one year after (late relapses/LR). To better understand genomic alterations underlying the delay of relapse, we identified copy number variations (CNVs) on 39 tumor samples from a homogeneous series of patients included in the Collaborative Trial in Relapsed Aggressive Lymphoma (CORAL) prospective study. To identify CNVs associated with ER or LR, we devised an original method based on Significance Analysis of Microarrays, a permutation-based method which allows control of false positives due to multiple testing. Deletions of *CDKN2A/B* (28%) and *IBTK* (23%) were frequent events in relapsed DLBCLs. We identified 56 protein-coding genes and 25 long non-coding RNAs with significantly differential CNVs distribution between ER and LR DLBCLs, with a false discovery rate < 0.05. In ER DLBCLs, CNVs were related to transcription regulation, cell cycle and apoptosis, with duplications of histone *H1T* (31%), deletions of *DIABLO* (26%), *PTMS* (21%) and *CK2B* (15%). In LR DLBCLs, CNVs were related to immune response, with deletions of *B2M* (20%) and *CD58* (10%), cell proliferation regulation, with duplications of *HES1* (25%) and *DVL3* (20%), and transcription regulation, with *MTERF4* deletions (20%). This study provides new insights into the genetic aberrations in relapsed DLBCLs and suggest pathway-targeted therapies in ER and LR DLBCLs.

## Introduction

Diffuse large B-cell lymphoma (DLBCL) is the most common subtype of non-Hodgkin lymphoma in adults [1]. The addition of the anti-CD20 monoclonal antibody rituximab to the chemotherapy CHOP (cyclophosphamide, adriamycin, vincristine and prednisone) has dramatically improved the outcome of patients with DLBCL [2, 3]. Despite this major advance a significant number of patients will experience treatment failure. At such a time, treatment is a real challenge and options are to be discussed regarding the eligibility or not for transplant and prognostic factors at relapse [4, 5]. One of the most important prognostic parameters in this context is the time of relapse [6, 7]. Two groups of patients are identified. The first group of early relapse (ER) is defined as relapse occurring within 1 year after the initial treatment. The second group of late relapse (LR) is defined as relapse occurring more than 1 year after the initial treatment. In the ER group, the overall survival (OS) is estimated at less than 17% at 3 years, while in the LR group, the OS is estimated at about 50% at 5 years [4].

The heterogeneous prognosis of patients with DLBCL reflects a deep biological diversity. DLBCL is composed of at least 2 molecular subtypes that differ in gene expression profile (GEP): germinal center B-cell like (GCB) and activated B-cell like (ABC), each with different prognosis [8]. The prognostic impact of this molecular classification has been reported in several independent series and different types of treatment at diagnosis as well as at relapse [9-11].

Recent advances in genomic technology have provided further understanding in the biology of DLBCL. Constitutive activation of the NF-κB pathway, owing to oncogenic mutations activating BCR (B-Cell Receptor) and toll-like receptor signaling, leads to cell proliferation and resistance to apoptosis. These constitutive activations, characterizing ABC DLBCLs, are related to somatic gain of function mutations and/or duplications affecting positive regulators of this pathway such as *CD79a* and *CD79b* [12], *MYD88* [13], *CARD11* [14], *TRAF2*, *TRAF5*, *MAP3K7/TAK1* and *TNFRSF11A/RANK* [15] or somatic inactivating mutations and/or deletions of negative regulators such as *TNFAIP3/A20* [15,16]. The GCB subtype is associated with abnormalities of epigenetic modifiers, with frequent bi-allelic point mutations leading to the loss of function of the histone lysine methyl-transferase *MLL2* [17,18], high frequency of gain-of-function mutations in the histone lysine methyl-transferase *EZH2* [19], inactivating mutations and deletions of the histone lysine acetyltransferases *CREBBP* and *EP300* [18], and mutations of their cofactor, *ME2FB* [17]. Constitutive activation of the PI3K/AKT/mTOR signaling pathway through* PTEN* deletions, *MiR17-92* locus amplifications and mutations of *PIK3CD, PIK3R1* and* mTOR* is another oncogenic mechanism commonly observed in GCB DLBCLs [20, 21]. Dysregulation of cell cycle is a common event in all DLBCLs, potentially owing to an increased expression of the anti-apoptotic factor *BCL2* [22-24], mono and bi-allelic deletions affecting the *CDKN2A/B* locus [22,25], point mutations and allele deletions leading to the loss of function of TP53 [26], or dysfunction of the transcription factor MYC, which has been described in 5 to 11% of *de novo* DLBCLs [27] and 17% of relapsed DLBCLs [28]. Constitutive activation of the NOTCH pathway through *NOTCH1* and *NOTCH2* somatic gain-of-function mutations or gain of copy numbers [29-31] cause an increased cell proliferation in B-cell lymphoma. *BCL6* expression beyond the germinal center reaction leads to increased tolerance to DNA breaks and blockade of plasmablastic differentiation [32, 33]. Importantly, escape to immune response through biallelic inactivations of *B2M* leading to defects of surface HLA class I molecules assembly [34], downregulation of surface HLA class II molecules expression due to breakpoints and mutations of *CIITA* [35] or deletions and truncating mutations of the *CD58* locus [34] represent another major player in lymphomagenesis in DLBCLs.

All these genomic studies have been conducted on *de novo* DLBCLs at diagnosis. Cellular functions and signaling pathway disruptions associated with relapsed DLBCLs remain largely unknown. Here we describe the number and the type of genetic alterations present in relapsed DLBCLs and make a comparison of Copy Number Variations (CNVs) between ER and LR DLBCLs to identify genetic alterations that are associated with the delay of relapse. To achieve this outcome, we designed an original method allowing the statistical assessment with the high level of confidence required when dealing with a very large set of candidates. Finally we evaluated the impact of relevant CNVs on gene expression.

## Results

### Landscape of large copy number variations in relapsed DLBCL

**Figure 1:**
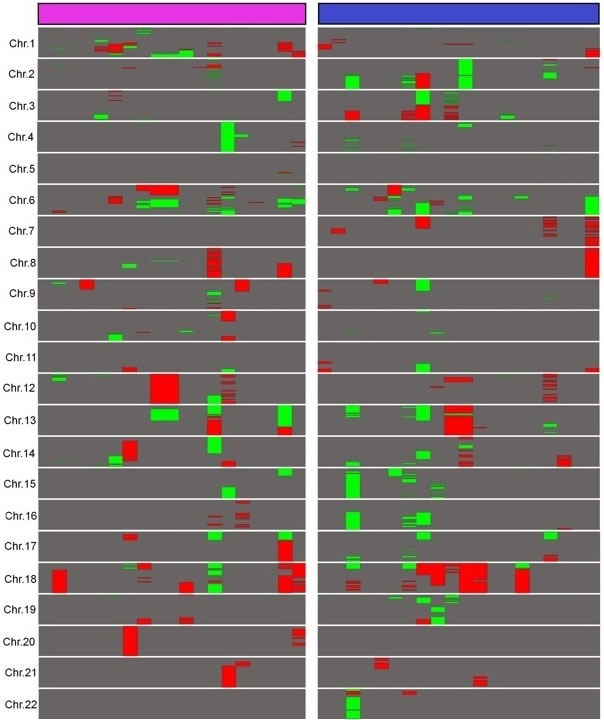
Landscape of large copy number variations in relapsed DLBCL A comparative representation of abnormalities longer than 2 Mb in ER and LR DLBCLs was built. Duplications are represented in red and deletions in green. ER DLBCLs (*n* = 19) are represented in purple and LR DLBCLs (*n* = 20) in blue. Chromosomes X and Y are not shown because of gender differences in copy numbers. This figure was obtained using Java Treeview 3.0.

CNVs ≥ 2 megabases (Mb) were counted to evaluate the complexity of structural aberrations of relapsed DLBCL (Figure 1). For the whole group, average CNVs per sample was 15.50 (ranging from 0 to 67). We found an equivalent number of duplications and deletions with an average 8.30 duplications per sample (0-43) and 7.20 deletions per sample (0-34; *p* = 0.60, Student's *t*-test). Chromosomes 1, 2, 3, 6, 12 and 18 were the most frequently altered, with 1.38 CNV per chromosome per sample on average while other chromosomes presented with an average of 0.45 CNV per chromosome per sample (*p* = 6x10^-4^).

In the ER group, the average CNVs per sample was 14.89. There was a wide heterogeneity within this group as samples presented with 0 to 67 CNVs ≥ 2 Mb. Proportions of duplications and deletions were comparable with an average 8.15 duplications (0-34) and 6.73 deletions per sample (0-33; *p* = 0.61). Chromosomes 1, 2, 6, 8, 12 and 18 were the most frequently altered, with an average of 1.58 CNV per chromosome per sample, whereas other chromosomes presented with an average of 0.34 CNV per chromosome per sample (*p* = 6x10^-3^).

In the LR group, the average CNVs per sample was 16.1. CNVs varied from 0 to 50 per sample within this group. Duplications and deletions were present in equivalent proportions with an average of 8.45 duplications per sample (0-43) and 7.65 deletions per sample (0-34), respectively (*p* = 0.81). Chromosomes 2, 3, 6, 7, 15, 18 were the most frequently altered, with an average of 1.55 CNV per chromosome, whereas remaining chromosomes presented with 0.42 CNV per chromosome on average (*p* = 7x10^-4^).

ER and LR DLBCLs samples were affected by an equivalent total CNVs number with an average of 14.89 and 16.11 CNVs per sample, respectively (*p* = 0.81). Comparing ER and LR for each chromosome revealed differential signatures on chromosome 1 only, with an average of 2.63 CNVs per sample in the ER group and 0.70 CNV per sample in the LR group (*p* = 6x10^-3^).

### Identification of genes differentially altered among the ER and the LR subgroups

After smoothing all the copy number probes using the sliding window technique, we observed that 0.38% of the values were below 1 and 1.53% above 3. This means that almost all losses and gains of copy numbers corresponded to heterozygous deletions and duplications, respectively.

**Figure 2:**
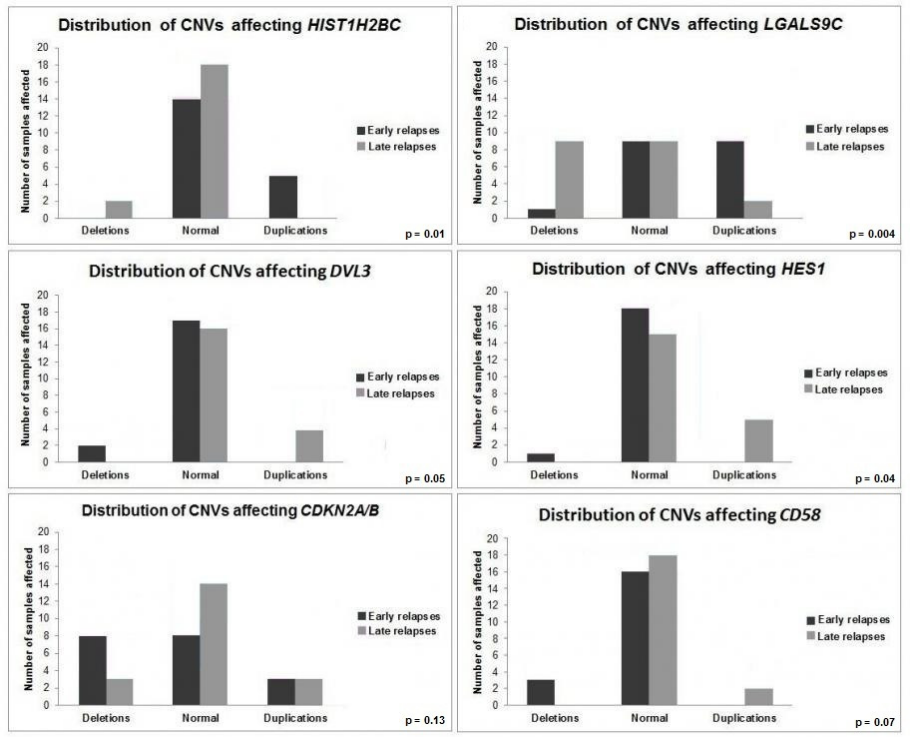
Genes differentially altered between ER DLBCLs and LR DLBCLs Comparison of CNV profiles between ER and LR DLBCLs has been performed at a genomic scale. *HIST1H2BC*, *LGALS9C, DVL3*, *HES1*, *CDKN2A/B* and *CD58* are genes with structural aberrations differentially distributed between ER and LR DLBCLs. ER DLBCLs (*n* = 19) are represented in dark grey and LR DLBCLs (*n* = 20) in light grey.

The use of Significance Analysis of Microarrays (SAM), a permutation-based method built on discriminating scores and their significance [36] identified 148 genes with significantly differential CNVs distribution between ER and LR DLBCLs with a false discovery rate (FDR) < 0.05. Among these genes were 56 protein coding genes (Table 1; Figure 2), 25 Long non-coding RNA (LncRNA), 22 antisense RNAs, 2 Small Nucleolar RNAs, 1 sense overlapping RNA, 1 miscellaneous RNA and 41 pseudogenes (Supplemental Table 1).

**Table 1:** Detailed list of the 56 protein coding genes presenting with differential CNVs distribution between ER and LR

Chr. Band	Gene	Gene full name	Description/function
1p21	*AMY2A*	Amylase, Alpha 2A	Member of the alpha-amylase family
1q44	*HNRPU*	Heterogeneous Nuclear Ribonucleoprotein U	pre-mRNA processing
1q44	*OR2T34*	Olfactory receptor, family 2, subfam. T, mb 34	G-protein-coupled olfactory receptor
1q44	*OR2AK2*	Olfactory receptor, family 2, subfam. AK, mb 2	G-protein-coupled olfactory receptor
2p11	*IGKV3D-7*	Immunoglobulin kappa variable 3D-7	BCR assembly
2q24	*GALNT5*	N-Acetylgalactosaminyltransferase 5	O-linked oligosaccharide biosynthesis
2q31	*NFE2L2*	Nuclear factor (erythroid-derived 2)-like 2	Transcriptionnal regulator
2q33	*SNORA1*	Small nucleolar RNA, H/ACA box 1	Novel snoRNA/Small nucleolar RNA
2q37	*MTERF4*	Mitochondrial Transcription Termination Factor 4	Regulator of mitochondrial gene expression
2q37	*UBE2F-SCLY*	UBE2F-SCLY Readthrough	MHC class I mediated antigen processing
2q37	*C2orf82*	Chromosome 2 Open Reading Frame 82	---
3q22	*PRR23B*	Proline Rich 23B	---
3q25	*AADAC*	Arylacetamide deacetylase	Microsomal arylacetamide deacetylase
3q27	*DVL3*	Dishevelled, dsh homolog 3	Regulation of cell proliferation
3q29	*CPN2*	Carboxypeptidase N, Polypeptide 2	Carboxypeptidase
3q29	*SENP5*	SUMO1/sentrin specific peptidase 5	Post-translational modification of proteins
3q29	*MUC20*	Mucin 20, Cell Surface Associated	Glycoprotein of mucous barrier
3q29	*HES1*	Hairy enhancer of split 1	Transcription factor
6p21	*CSNK2B/CK2B*	Casein kinase 2, beta subunit	Regulation of programmed cell death
6p21	*BTNL2*	Butyrophilin-Like 2	Negative regulation of T-cell proliferation
6p21	*CCHCR1*	Coiled-Coil Alpha-Helical Rod Protein 1	Regulation of keratinocyte proliferation
6p21	*HIST1H1T*	Histone Cluster 1, H1t	Epigenetic regulation of transcription
6p21	*HLA-DRA*	MHC complex, Class II, DR Alpha	Antigen presentation
6p22	*HIST1H2BC*	Histone Cluster 1, H2bc	Epigenetic regulation of transcription
6p22	*HIS1H2AK*	Histone Cluster 1, H2ak	Epigenetic regulation of transcription
6p22	*KAAG1*	Kidney Associated Antigen 1	---
6p25	*SERPINB1*	Serpin Peptidase Inhibitor, Class B , Mb1	Proteinase inhibition
6q13	*KHDC1*	KH homology domain containing 1	Predicted membrane protein
7p22	*GPR146*	G Protein-Coupled Receptor 146	G protein coupled receptor
7q11	*ABHD11*	Abhydrolase domain containing 11	---
7q11	*STX1A*	Syntaxin 1A	Docking of synaptic vesicles
11p15	*ART5*	ADP-Ribosyltransferase 5	ARG-specific ADP-ribosyltransferase
12p13	*PRH1*	Proline-Rich Protein HaeIII Subfamily 1	Inhibition of crystal calcium phosphates
12p13	*C1R*	Complement Component 1, R Subcomponent	Complement activation
12p13	*PTMS*	Parathymosin	Inhibition of linkage of H1 to chromatin
12q24	*DIABLO*	Diablo, IAP-Binding Mitochondrial Protein	Apoptosis promotion
12q24	*LRRC43*	Leucine Rich Repeat Containing 43	---
13q13	*EXOSC8*	Exosome Component 8	Transcriptionnal regulation
14q11	*OR4Q3*	Olfactory receptor, family 4, subfam. Q, mb 3	G-protein-coupled olfactory receptor
14q11	*TRAV-26-1*	T Cell Receptor Alpha Variable 26-1	TCR assembly
14q11	*TRAJ10*	T Cell Receptor Alpha Joining 10	TCR assembly
14q11	*TRAJ13*	T Cell Receptor Alpha Joining 13	TCR assembly
14q12	*RP11-468E2.6*	---	---
14q32	*IGHVIII-2-1*	Immunoglobulin Heavy Variable (III)-2-1	BCR assembly
14q32	*IGHVIII-16-1*	Immunoglobulin Heavy Variable (III)-16-1	BCR assembly
14q32	*DCAF11*	DDB1 And CUL4 Associated Factor 11	Assembly of the DDB1–CUL4A ubiquitin ligase
15q15	*CATSPER2*	Cation Channel, Sperm Associated 2	Sperm motility and male fertility
16p13	*NARFL*	Nuclear prelamin A recognition factor-like	Modulation of hypoxia-inducible factor-1*α* activity
16q22	*HAS3*	Hyaluronan Synthase 3	Synthesis of glycosaminoglycan hyaluronan
16q22	*CDH16*	Cadherin 16	Ca2+-dependent glycoprotein
16q22	*CLEC18B*	C-Type Lectin Domain Family 18, Member B	---
17p11	*LGALS9C*	Lectin, Galactoside-Binding, Soluble, 9C	Regulation of immune response
19p13	*LRG1*	Leucine-rich alpha-2-glycoprotein 1	Signal transduction, and cell adhesion
19p13	*ZNF414*	Zinc Finger Protein 414	Transcriptional regulation
19p13	*AC008686.1*	---	Uncharacterized protein
22q13	*ADSL*	Adenylosuccinate lyase	*De novo* purine biosynthesis

The SAM method allowed identification of 148 genes with differentially distributed genomic alterations between ER and LR DLBCLs. The 56 protein coding genes are presented in this table.

ER DLBCLs were characterized by frequent duplications affecting *HIST1H1T* (6/19 samples, 31%), *HIST1H2BC* (5/19 samples, 26%), *HIST1H2AK* (5/19 samples, 26%), and *PTMS* (4/19 samples, 21%), implicated in histone-related regulation of transcription. We found a high number of duplications of *AIRN* (4/19 samples, 21%), a LncRNA interacting with the chromatin remodeling complex G9a. Deletions of *HNRNPU* (4/19 samples, 21%), implicated in pre-mRNA processing and alternative splicing, were also present. In addition, deletions of programmed-cell-death implicated genes *DIABLO* (5/19 samples, 26%) and *CSNK2B/CK2B* (3/19 samples, 15%), were identified. Duplications of *LGALS9C* (9/19 samples, 47%), deletions of *BTNL2* (5/19 samples, 26%) and *HLA-DRA* (4/19 samples, 21%) were also revealed. A significant fraction of samples was affected by duplications of *CCHCR1* (6/19 samples, 31%), a regulator of keratinocyte proliferation.

LR DLBCLs were characterized by a high number of duplications affecting *DVL3* (4/20 samples, 20%), a regulator of cell proliferation. We found frequent loss of copy number for *LGALS9C* (9/20 samples, 45%), *GALNT5* (4/20 samples, 21%), implicated in O-linked oligosaccharide biosynthesis, *ADSL* (3/20 samples, 15%), involved in *de novo* purine biosynthesis and *ART5* (4/20 samples, 20%), an ADP-ribosyltransferase. Genes implicated in BCR (*IgHV*,* IgKV*) and T-Cell Receptor (TCR) assembly *(TRAJ* and *TRAV*) and transcriptional regulation, such as *NFE2l2*, *MTERF4*, *HES1*, *EXOSC8*, *NARFL* and *ZNF414*, were affected by either duplication or deletion in at least 20% of LR DLBCL samples.

**Figure 3:**
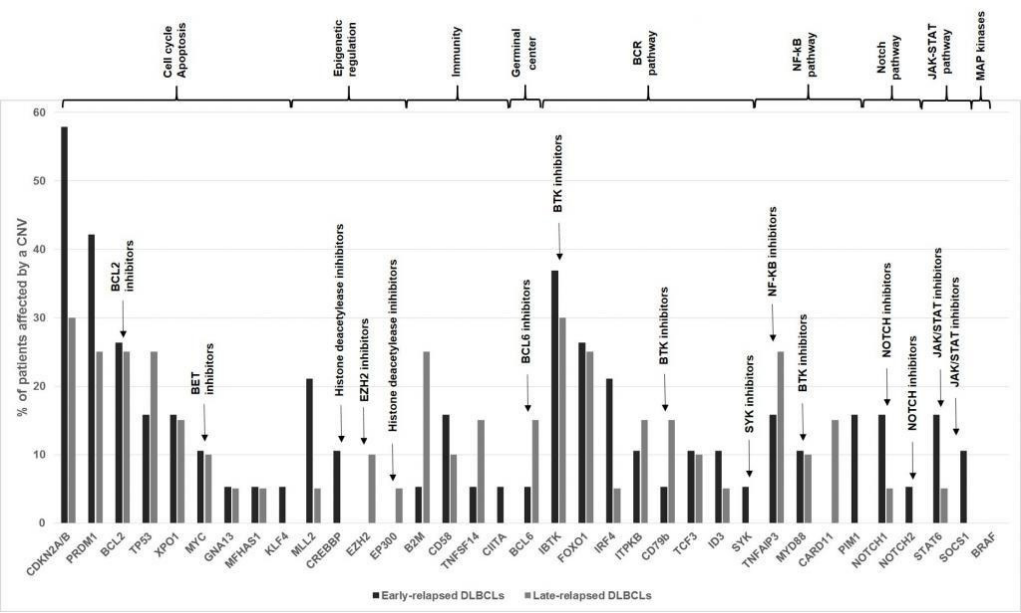
Genomic alterations of the 36 genes set involved in lymphomagenesis Comparative distribution of CNVs among ER and LR DLBCLs is shown. Genes are grouped according to key dysregulated pathways in DLBCLs. ER DLBCLs (*n* = 19) are colored in dark grey and LR DLBCLs (*n* = 20) in light grey. CNV: Copy Number Variation.

Among the subset of 36 genes selected for their implication in lymphomagenesis, the 10 most frequently altered were *CDKN2A/B* (43%)*, IBTK* (33%)*, PRDM1* (33%)*, FOXO1* (25%)*, BCL2* (25%)*, TP53* (20%)*, TNFAIP3* (20%)*, XPO1* (15%)*, B2M* (15%) and* CD58* (12%)(Figure 3). Cell cycle regulation and apoptosis were the most represented of affected pathways, with a high number of alterations encompassing *CDKN2A/B*, *PRDM1*, *BCL2*, *TP53* and *XPO1*. Deletions of *CDKN2A/B* and *IBTK* were identified in 28% and 23% of DLBCL samples, respectively.

ER DLBCLs harbored frequent deletions of *CDKN2A/B* (8/19 ER samples, 42% *versus* 3/20 LR samples, 15%), *PIM1* (3/19 ER samples, 15% *versus* 0/20 LR sample, 0%), *MLL2* (4/19 ER samples, 21% *versus* 1/20 LR sample, 5%) and *CREBBP* (2/19 ER samples, 10% *versus* 0 LR sample). In LR DLBCLs, we identified CNVs affecting *B2M* (5/20 LR samples, 25% *versus* 1/19 ER samples, 5.26%) (*p* = 0.1), deletions of *B2M* being restricted to LR DLBCLs, and *CD58* (deletions in 2/20 LR samples, 10% *versus* duplications in 3/19 ER samples, 15.7%) (*p* = 0.07).

When we compared CNVs distribution among ER and LR samples, we did not find any statistically significant difference. We also did not observe any correlation between CNVs distribution and the GC/ABC subtype (data not shown).

### Evidence on recurrent events in relapsed DLBCLs

**Figure 4:**
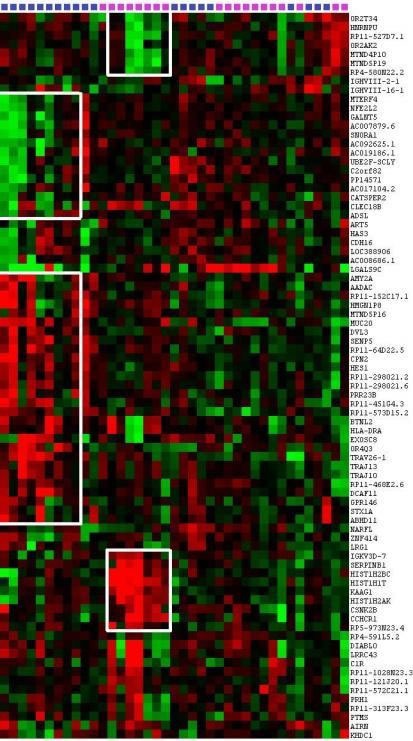
Evidence for recurrent events in relapsed DLBCLs Hierarchical clustering of 56 protein-coding genes CNVs and 25 LncRNAs CNVs identifies events associated with ER and LR DLBCLs. The heatmap shows recurrent CNVs in DLBCL patients and gives evidence of genetic aberration occurrences in ER and LR DLBCLs. In ER DLBCLs, *HIST1H2BC*, *HIST1H1T*, *HIS1H2AK*, *CSNK2B/CK2B*, *KAAG1*, *CCHCR1*, and *IgKV3D-7* are co-amplified in the same patients. Accordingly, in LR DLBCLs, *AMY2A*, *AADAC*, *MUC20*, *DVL3*, *SENP5*, *CPN2*, and *HES1* are co-amplified in the same patients. This figure was obtained using Cluster 3.0 and Java Treeview 3.0.

We performed a hierarchical clustering on 56 protein-coding genes and 25 LncRNA CNVs to identify events recurrently associated with ER and LR DLBCLs (Figure 4). The resulting heatmap shows recurrent CNVs in DLBCLs and defines three groups of patients.

The first group is homogeneous and comprises 7 ER samples presenting with correlated amplifications of *HIST1H2BC*, *HIST1H1T*, *HIS1H2AK*, *CSNK2B/CK2B*, *KAAG1*, *CCHCR1*, SERPINB1 and *IgKV,* as well as correlated deletions of *HRNPU* and four LncRNAs: *RP11-527D7.1*, *MTND4P10*, *MTND5P19* and *RP4-580N22.2*.

The second group is composed of 9 LR DLBCLs showing correlated amplifications of *AMY2A*, *AADAC*, *MUC20*, *DVL3*, *SENP5*, *CPN2*, and *HES1* as well as correlated deletions of* MTERF4*, *NFE2L2*, *GALNT5*, *ADSL* and three LncRNAs: *AC007879.6*, *AC092625.1*, *and AC019186.1*.

Remaining ER and LR samples constituted a third group without specific CNV profiles and no recurrent events.

### Impact of CNVs on gene expression

**Figure 5:**
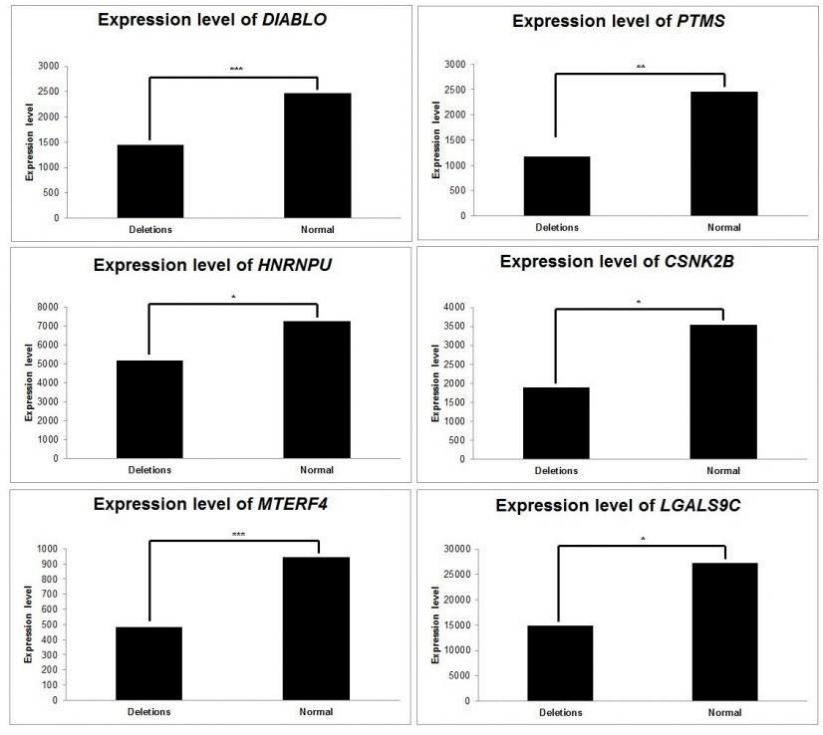
Consequences of relevant copy number variations on gene expression Average gene expression was computed, according to relevant CNVs. CNVs affecting* DIABLO, PTMS, HNRNPU, CSNK2B, MTERF4 and LGLAS9C* have subsequent consequences on the expression levels of those genes. *: *p* < 5 x10^-2^; **: *p* < 10^-2^; ***: *p* < 10^-3^.

We examined the impact of CNVs on average gene expression level (AGEL) (Figure 5).

In ER DLBCLs, deletions of *DIABLO* were related to a decrease in its AGEL, with a fold change of 0.58 between deleted and normal samples (*p* = 5x10^-4^, Student's t-Test). We observed the same correlation between deletions of the genes and decrease in their expression level for *PTMS, HNRPU, and CSNK2B*. Deletions of these three genes resulted in a decrease in their respective AGELs, with a fold change of 0.47, 0.71, and 0.53, respectively (*p* = 0.01, 0.02 and 0.03).

In LR DLBCLs, deletions of *MTERF4* were related to a decrease in its AGEL, with a fold change of 0.51 between deleted and normal samples (*p* = 8x10^-7^). Accordingly, deletions of *LGALS9C* led to a decrease in AGEL compared to normal samples (fold change = 0.54; *p* = 0.05).

In the set of 36 genes, deletions of *CDKN2A/B* were related to a decrease in AGEL (fold change = 0.31; *p* = 0.07). Deletions of *CD58* were also related to a decrease in AGEL (fold change=0.65; *p* = 0.08).

## Discussion

Understanding the relationship between tumor biology and outcome is important for identifying molecular targets and may yield more effective therapies for DLBCLs. Almost all genomic studies reported to date were performed on *de novo* DLBCLs at diagnosis and little is known about structural aberrations of refractory/relapsed DLBCLs. Here we analyzed one of the few series of relapsed DLBCLs published to date [37, 38], composed of 39 samples from the Collaborative Trial in Relapsed Aggressive Lymphoma (CORAL), a unique and well-documented prospective study on relapsed DLBCLs [7]. Studying this thirty-nine-patient cohort allowed us to make strong statements since we devised and used for the first time a robust, comprehensive, unsupervised analysis method to analyze CNVs. This method makes use of SAM, a statistical technique originally designed for gene expression data analysis [36]. This permutation-based method allowed the discovery of a list a gene containing less than 5% of false positives despite multiple testing, independent from data distribution. Applied to oligonucleotide microarrays covering the entire genome, the method allowed us to delineate statistically validated CNVs with great precision and locate structural aberrations linked with ER and LR DLBCLs. Other published algorithms such as GISTIC [39], GEDI [40], or ComFocal [25] are designed to identify recurrent regions of deletions or duplications, which is not the purpose of this work. Here we aimed at identifying CNVs strongly associated with ER or LR, which is the main reason motivating the development of this original method.

**Figure 6:**
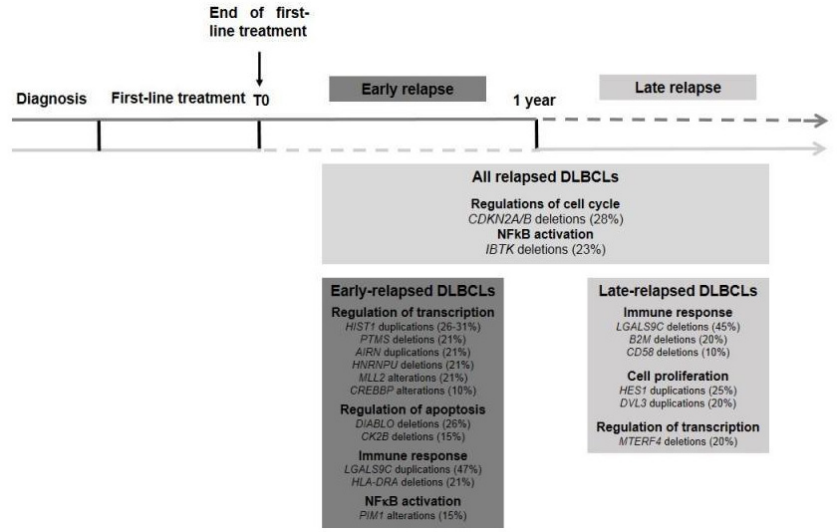
Key oncogenic pathways in relapsed DLBCLs Structural abnormalities related with relapsed DLBCLs are shown, with distinction between ER and LR DLBCLs. ER DLBCLs are preferentially associated with dysregulation of gene transcription, cell cycle and apoptosis. LR DLBCLs are rather associated with dysregulation of immune response, cell proliferation and regulation of transcription.

ER and LR DLBCLs display a comparable CNV landscape, with a high degree of heterogeneity within each group. In relapsed DLBCLs, the most frequently altered genes were related to cell cycle regulation and apoptosis, regulation of transcription, immune response and NF-κB pathway (Figure 6). Among genes associated with lymphomagenesis, CNVs affecting *CDKN2A/B* locus (42% of samples) were the most frequent, with a majority of deletions (28% of samples). CDKN2A is a cycline-dependent kinase restraining cellular proliferation. These results are consistent with previous reports describing 30-35% of *CDKN2A* deletions in primary DLBCLs, predominantly in ABC subtype, with subsequent increase in cell proliferation and poorer outcome [22, 41, 42]. We also observed a high rate of *IBTK* aberrations (33% of samples), mainly deletions (23% of samples). IBTK (Inhibitor of Burton Tyrosine Kinase) is a BTK binding protein which inhibits BTK's kinase activity and disrupts BTK-mediated calcium mobilization leading to a decrease in BTK-induced activation of the anti-apoptotic NF-κB pathway [43]. A recent study reported a high rate of complete or partial responses and long-lasting remissions with ibrutinib treatment in relapsed/refractory DLBCLs, essentially in ABC subtype [44]. In this work, we did not observe any correlation between CNVs and the GC/ABC subtype. These results are in keeping with a recently published study showing (i) an absence of correlation between known driver mutations in lymphomagenesis and response to therapy, and (ii) an equivalent distribution of genomic events predicting survival across GC and ABC subtypes [37].

When we compared ER and LR DLBCLs at the gene scale, aberrant genomic profiles were distinct on 56 protein-coding genes, with a subsequent effect on gene expression levels. In some cases, we were unable to demonstrate a statistically significant effect on gene expression. This can be explained by intronic localization of CNVs.

First, structural aberrations of cell cycle and apoptosis regulators were rather associated with ER DLBCLs, with deletions of *DIABLO/Smac*, a promoter of apoptosis which allows activation of caspases by binding to inhibitor of apoptosis proteins [45], and deletions of *CSNK2B/CK2B*, the regulatory subunit of protein kinase CK2, involved in a wide number of cellular processes, such as regulation of programmed cell death, cell division and proliferation, DNA damage repair, gene transcription and protein translation. CK2 increased protein expression and enzymatic activity has been described to positively regulate the PI3K pathway and STAT3 and NF-κB signaling in hematological malignancies [46]. We hypothesize that deletions of the *CSNK2B/CK2B* regulatory subunit with subsequent decrease in gene expression level result in increased enzymatic activity. LR DLBCLs were associated with duplications of *DVL3*, a member of the disheveled protein family which has been described to promote cell growth in ALK-positive anaplastic large cell lymphoma [47].

Second, CNVs encompassing genes related to immune response included duplications of *LGALS9C* identified in 47% of ER DLBCLs. LR DLBCLs were associated with partial deletions and duplications of *TRAJ* and* TRAV*, both implicated in TCR assembly. This suggests infiltration of the tumor by T-cell lymphocytes as part of the immune response. Deletions of *B2M* (20%) and *CD58* (10%) were restricted to LR DLBCLs. Deletions and duplications of HLA related genes, were observed frequently in our cohort and are most likely related to HLA system polymorphism. Losses in *IgHV* and *IgKV* clusters are presumably a sign of BCR rearrangement and cannot be considered as recurrent lesion in tumor samples. We made the choice to consider all CNVs, including those that are described in databases of genomic variants. Limiting our analysis to acquired lesions may avoid constitutional abnormalities that are prone to foster lymphoma development.

Third, dysregulation of transcription through altering events in genes encoding regulators of chromatin compaction and DNA replication were associated with ER DLBCLs. Duplications of the linker histone H1 gene and/or deletions of its regulatory partner *PTMS* [48] were found in 21 to 31% of cases. This observation is consistent with a previous report, where mutations of the linker histone H1 gene have been described as a key evolutionary process governing transformation of follicular lymphoma into DLBCL [49]. LR DLBCLs were linked with deletions of *MTERF4*, a mitochondrial transcription regulator [50], in 20% of cases. LncRNA are nucleic acids often longer than 2 kb involved in the regulation of transcription through epigenetic modifications by recruiting chromatin remodeling complexes to various genomic loci [51]. *AIRN (*Air noncoding RNA) was of particular interest as it was deleted in 20% of ER DLBCLs. This LncRNA plays a role in epigenetic regulation of transcription by cooperating with the histone methyl transferase G9a [52]. Duplications of *HES1*, a downstream effector of the NOTCH pathway associated with Fanconi anemia [53] and blast crisis transformation in chronic myelogenous leukemia [54] were restricted to LR samples (25%).

Even though DLBCL is a heterogeneous disease, our method allowed the identification of structural aberrations that are statistically associated with ER and LR. Moreover, hierarchical clustering of CNVs identified three groups of patients. Two groups (the first one composed of ER samples and the second one of LR samples) were well separated in homogeneous clusters with common deletions and duplications. The remaining samples formed a mixed group of ER and LR samples with no recurrent CNV. This result gives evidence of a continuum between ER and LR DLBCLs. Identifying two groups of samples with correlated CNVs profiles demonstrates the statistical significance of our results, since these correlated groups could not be identified by chance.

Our original method unraveled genomic aberrations typical of the “LR signature”, which includes genomic aberrations encompassing genes related to immune response, cell proliferation and regulation of transcription. The “ER signature” includes events leading to disruption of cell cycle, apoptosis and gene transcription and provides a structural basis for selective growth advantage and chemotherapy resistance leading to a worse prognosis. This is of great importance since it may be a starting point for further studies and supports the rationale for targeted therapy. Even if this study needs to be confirmed on a larger cohort of patients, it provides new insights into the genetic complexity and dysregulated pathways in relapsed DLBCLs, suggesting the possibility of individualized targeted therapy for those patients with poor prognosis.

## Materials and methods

### Patient cohort

The phase III CORAL prospective study included 396 patients presenting with relapsed/refractory DLBCL after first-line treatment with R-CHOP (rituximab, cyclophosphamide, adriamycin, vincristine and prednisone). These patients were randomly assigned to receive a salvage chemotherapy regimen, either rituximab, ifosfamide, carboplatin and etoposide (R-ICE) or rituximab, dexamethasone, cytarabine, and cisplatin (R-DHAP). Patients who responded to the chemotherapy were submitted to high dose therapy and autologous stem cell transplant (ASCT). After ASCT, the trial compared rituximab treatment every 2 months for 1 year with observation alone. The initial results revealed no significant difference in outcome. Several factors did affect survival, including ER, the international prognostic index at relapse, and prior exposure to rituximab [7].

From these 396 patients with relapsed/refractory DLBCL, frozen lymph node biopsies were available from a homogeneous group of 39 patients. A part of tumors were sampled at diagnosis, before first-line treatment, and the others were sampled at relapse, before salvage chemotherapy. Thus, among the 19 tumor samples composing the ER group, 11 were sampled at diagnosis, and 8 at relapse. Among the 20 tumor samples from the LR group, 9 were sampled at diagnosis and 11 at relapse. Molecular characterization regarding GCB DLBCLs and ABC DLBCLs could be determined with GEP in 30 cases. Characteristics of the patients are listed in Table 2. Procedures used were in accordance with the Helsinki Declaration of 1975 (as revised in 1983).

**Table 2:** Characteristics of the cohort of 39 relapsed DLBCLs

N°	Sex	Age at diagnosis	IPI at diagnosis	First-line chemotherapy regimen	Delay of relapse (months)	ER/LR	Subtype	Rearrangements			Sample
								*MYC*	*BCL2*	*BCL6*	
1	Male	62	3	CHOP	11	ER	GC	No	1	2	Diagnosis
2	Female	20	1	CHOP	6	ER	ABC	No	2	2	Diagnosis
3	Female	60	4	CHOP	6	ER	ABC	No	1	2	Diagnosis
4	Female	60	4	ACVB	8	ER	NA	No	2	2	Diagnosis
5	Male	59	0	CHOP	3	ER	GC	No	1	2	Relapse
6	Male	28	0	ACVB	9	ER	NA	No	2	2	Relapse
7	Male	43	1	CHOP	6	ER	ABC	NA	NA	NA	Diagnosis
8	Male	41	1	CHOP	11	ER	NP	NA	NA	NA	Relapse
9	Female	38	3	CHOP	5	ER	ABC	No	2	2	Diagnosis
10	Male	46	2	CHOP	0	ER	GC	No	1	NA	Diagnosis
11	Male	34	2	CHOP	2	ER	GC	NA	NA	NA	Relapse
12	Male	54	0	ACVB	9	ER	GC	No	1	2	Relapse
13	Male	57	1	CHOP	0	ER	GC	Yes	1	2	Diagnosis
14	Male	57	1	CHOP	0	ER	GC	Yes	1	2	Relapse
15	Male	57	2	ACVB	2	ER	NP	No	2	NA	Diagnosis
16	Male	32	2	CHOP	0	ER	GC	No	2	2	Relapse
17	Female	35	2	ACVB	7	ER	ABC	NA	NA	NA	Diagnosis
18	Female	35	2	ACVB	7	ER	ABC	No	2	2	Relapse
19	Male	33	0	ACVB	11	ER	GC	No	2	2	Diagnosis
20	Male	29	2	ACVB	82	LR	GC	NA	NA	NA	Relapse
21	Male	41	0	CHOP	148	LR	NA	NA	NA	NA	Relapse
22	Male	42	2	ACVB	29	LR	NA	Yes	2	2	Diagnosis
23	Male	41	1	ACVB	155	LR	ABC	No	2	2	Relapse
24	Male	59	0	ACVB	20	LR	GC	No	1	2	Relapse
25	Male	53	0	ACVB	50	LR	GC	No	2	2	Diagnosis
26	Male	53	0	ACVB	50	LR	GC	No	2	2	Relapse
27	Female	58	2	ACVB	60	LR	ABC	NA	1	1	Relapse
28	Male	46	0	ACVB	40	LR	ABC	NA	NA	NA	Diagnosis
29	Male	33	0	ACVB	109	LR	ABC	No	2	2	Relapse
30	Male	43	0	ACVB	103	LR	ABC	No	2	2	Diagnosis
31	Female	38	1	CHOP	62	LR	NA	No	2	NA	Diagnosis
32	Male	43	0	CHOP	158	LR	GC	Yes	1	NA	Diagnosis
33	Male	62	2	CHOP	21	LR	NA	No	1	2	Diagnosis
34	Male	56	0	ACVB	29	LR	GC	No	2	2	Diagnosis
35	Male	56	0	ACVB	29	LR	NA	No	2	2	Relapse
36	Male	45	0	CHOP	153	LR	GC	No	1	2	Relapse
37	Male	26	0	CHOP	116	LR	GC	No	2	2	Relapse
38	Male	58	2	CHOP	19	LR	NA	NA	NA	NA	Diagnosis
39	Male	NA	NA	NA	NA	LR	NA	No	2	1	Relapse

IPI: International prognostic index; CHOP: cyclophosphamide, adriamycin, vincristine and prednisone; ACVB: adriamycin, cyclophosphamide, vindesine, bleomycin; ER: Early Relapse; LR: Late Relapse; GC: Germinal Center B; ABC: Activated B-cell; NA: Non Available; NP: Non Predictable.

### Copy number analysis

CNVs of the 39 samples were determined using the Affymetrix SNP 6.0 platform (Affymetrix, Santa Clara, CA, USA), which interrogates 1 800 000 copy number probes distributed evenly along the human genome (mean interval: 1.8 kilobases) [55]. The 1 800 000 probes were annotated with home-made tools querying the Ensembl 77^th^ version with Human Genome HG37 MYSQL database available at ftp.ensembl.org/pub/release-77/mysql/. Total copy numbers were computed using the Bioconductor crlmm package [56]. A sliding window (length = 11 probes) representing the signal median value was used to smooth artifactual data.

This strategy allowed CNV discovery at high resolution and identification of genes frequently altered in relapsed DLBCLs. Duplications were defined by a copy number ≥2.5, deletions were defined by a copy number ≤1.5 and copy numbers between 1.5 and 2.5 were considered normal, according to an already published method [22]. We used Java Treeview 3.0 [57] to visualize the results at a genomic scale.

### Identification of differential genomic alterations between ER and LR

We aimed at identifying genes affected by differentially distributed CNVs between ER and LR DLBCL patients, *ie* cases where deletions and duplications are associated preferentially with ER or LR. Given the large number of statistical tests, we used a permutation-based analysis to reduce false-positive results due to multiple testing. Our method was based on the widely used technique called Significance Analysis of Microarrays (SAM) [36]. SAM is a non-parametric test that avoids unjustified assumptions over gene distribution and gene independence. SAM was originally designed to determine whether changes in gene expression are experimentally significant. As claimed by the authors, SAM can be adapted for other experimental types of data. For our purpose, one needs to define a measure of the strength of the relationship between the CNV distribution (loss of copy number, normal copy number and gain of copy number) and ER or LR. Many statistics such as Pearson's chi-squared test, and other measures such as the Kullback-Leibler divergence, are only defined in case of non-null frequencies. Therefore we preferred to select the Hellinger distance *H*(*P*,*Q*) which quantifies the difference between probability distributions *P* = (*p*1*,…, p*k) and *Q* = (*q*1*,…, q*k). The Hellinger distance is defined as:

$$H\left(P,Q\right)=\frac{1}{\sqrt{2}}\;\sqrt{\overset{k}{\underset{i=1}{\sum }}{\left(\sqrt{pi}\;-\;\sqrt{qi}\right)}^{2}}$$

The lower bound of *H*(*P*,*Q*) = 0 corresponds to *P = Q* and its upper bound *H*(*P*,*Q*) = 1 implies that *p*i*q*i = 0 " *i* Î [1,*k*]. In our method, P and Q represent the probability distributions over the ER and LR DLBCL copy numbers, expressed in three discrete states (deletion, normal, duplication).

Genes were considered positive when the FDR was inferior to 5%. For a FDR of 5%, our algorithm can be stated as: (i) compute the observed Hellinger distances for all the probes mapping to gene *g*i. The distance associated to *g*i is defined as the mean of the probe distances. (ii) form an observed vector *H*i by ordering the observed gene distances. (iii) perform *B* = 20 random permutations over the patient labels and compute the corresponding expected null gene distances. Order the B distance vectors and compute the corresponding mean expected vector *H*Ei. (iv) plot the observed vector against the expected null vector (Supplemental Figure 1). (v) set a threshold D = max(*H*i - *H*Ei) (vi) define the significant genes verifying *H*i - *H*Ei ^3^ D (vii) estimate the FDR. This value can be computed as the average of genes called significant from all the *B* permutations. (viii) if FDR > 0.05, decrease the threshold D and repeat steps 6 and 7.

Then, the aim of this method is not to provide an individual p-value for each gene of the genome but to identify of a list of genes which CNVs distribution is differential between ER and LR, with systematic correction of multiple testing and less than 5% of false positives.

SNP microarray data acquired on Affymetrix SNP 6.0 platform can be accessed online on Gene Expression Omnibus (GSE73791).

### Selected set of genes implicated in lymphomagenesis

We also explored a subset of 36 genes selected from literature and already known to play a role in lymphomagenesis through the dysregulation of key cellular pathways [58]. *CDKN2A, CDKN2B, PRDM1, TP53, BCL2, XPO1, MYC, GNA13, MFHAS1* and *KLF4* were chosen for their implication in cell cycle and apoptosis; *MLL2, CREBBP, EZH2* and* EP300* for their role in epigenetic regulation; *B2M, CD58, TNFSF14* and* CIIT*A for their implication in dysregulation of immunity and escape to immune response; *BCL6* for its role in germinal center reaction; *IBTK, FOXO1, IRF4, ITPKB, CD79b, TCF3, ID3* and* SYK* for their implication in the BCR pathway; *TNFAIP3, MYD88, CARD11* and* PIM1,* for their implication in constitutive activation of NF-κB pathway; *NOTCH1* and* NOTCH2* for their association with NOTCH pathway disruption; *STAT6* and SOCS1 implicated in JAK-STAT pathway; *BRAF* for its role in MAP kinase pathway.

### CNV impact on gene expression through integrative genomics

Gene expression profiles were available for 30 out of the 39 patients. These data were acquired on an Agilent Whole Human Genome microarray 4x44K platform (Agilent technologies, Santa Clara, CA, USA). Gene expression levels for each sample were integrated with CNVs. The impact of deletions and duplications of the selected genes on their own expression level was evaluated by comparing the average gene expression levels of deleted, duplicated and normal samples using Student's t-test.

## SUPPLEMENTARY MATERIALS FIGURE AND TABLE


