# Supplementary material for: Relapsed diffuse large B-cell lymphoma present different genomic profiles between early and late relapses
Source: Oncotarget. 2016 Jun 2;7(51):83987–4002. doi: 10.18632/oncotarget.9793 (PMC5356640; doi:10.18632/oncotarget.9793)
Supplement: Supplementary file 1 [file oncotarget-07-83987-s001.pdf]

Relapsed diffuse large B-cell lymphoma present different genomic profiles between early and late relapses

Supplementary Material

Supplemental table 1. List of the 148 genes with differential CNVs distribution among early-relapsed and late-relapsed DLBCLs.

Chr. Band	Gene	Gene full name	Description/function
1p21	*AMY2A*	Amylase, Alpha 2A	Member of the alpha-amylase family
1p35	*RP4-591L5.2*	---	LncRNA
1p35	*RP1-65J11.5*	---	Antisense RNA
1q23	*RP11-367J7.4*	---	Pseudogene
1q32	*RP11-168O16.1*	---	Antisense RNA
1q42	*RP4-580N22.2*	---	LncRNA
1q42	*MTND5P19*	MT-ND5 Pseudogene 19	LncRNA
1q42	*MTND4P10*	MT-ND4 Pseudogene 10	LncRNA
1q42	*MTND6P14*	MT-ND6 Pseudogene 14	Pseudogene
1q42	*RP5-827C21.2*	---	Antisense RNA
1q43	*RP11-193H5.2*	---	Pseudogene
1q43	*RP11-567G24.1*	---	Pseudogene
1q43	*RP11-527D7.1*	---	LncRNA
1q43	*RNU6-1139P*	---	snRNA
1q44	*HNRNPU-AS1*	HNRNPU Antisense RNA 1	Antisense RNA
1q44	*FABP7P1*	Fatty acid binding protein 7, brain pseudogene 1	Pseudogene
1q44	*RNU6-947P*	RNA, U6 Small Nuclear 947, Pseudogene	Pseudogene
1q44	*HNRPU*	Heterogeneous Nuclear Ribonucleoprotein U	pre-mRNA processing
1q44	*OR2T34*	Olfactory receptor, family 2, subfam. T, mb 34	G-protein-coupled olfactory receptor
1q44	*OR2AK2*	Olfactory receptor, family 2, subfam. AK, mb 2	G-protein-coupled olfactory receptor
2p11	*IGKV3D-7*	Immunoglobulin kappa variable 3D-7	BCR assembly
2q23	*AC079790.2*	---	Antisense RNA
2q24	*GALNT5*	N-Acetylgalactosaminyltransferase 5	O-linked oligosaccharide biosynthesis
2q24	*AC092625.1*	---	LncRNA
2q24	*AC019186.1*	---	LncRNA
2q24	*AC009227.2*	---	Antisense RNA
2q31	*EXTL2P1*	Exostoses (multiple)-like 2 pseudogene 1	Pseudogene
2q31	*NFE2L2*	Nuclear factor (erythroid-derived 2)-like 2	Transcriptionnal regulator
2q32	*RN7SL267P*	RNA, 7SL, cytoplasmic 267	Pseudogene
2q33	*RNU6-31P*	RNA, U6 Small Nuclear 31	snRNA
2q33	*AC007163.9*	---	Pseudogene
2q33	*MTND4P29*	MT-ND4 Pseudogene 29	Pseudogene
2q33	*MTND5P31*	MT-ND5 Pseudogene 31	Pseudogene
2q33	*SNORA1*	Small nucleolar RNA, H/ACA box 1	Novel snoRNA
2q33	*AC007879.6*	---	LncRNA
2q34	*MEAF6P1*	MYST/Esa1-associated factor 6 pseudogene 1	Pseudogene
2q35	*AC053503.11*	---	Antisense RNA
2q35	*AC010136.2*	---	Antisense RNA
2q35	*CXCR2P1*	Chemokine Receptor 2 Pseudogene 1	Pseudogene
2q37	*AC017104.2*	---	LncRNA
2q37	*PP14571*	---	LncRNA
2q37	*MTERF4*	Mitochondrial Transcription Termination Factor 4	Regulator of mitochondrial gene expression
2q37	*UBE2F-SCLY*	UBE2F-SCLY Readthrough	MHC class I mediated antigen processing
2q37	*C2orf82*	Chromosome 2 Open Reading Frame 82	---
2q37	*AC104667.3*	---	Antisense RNA
3p22	*AC011816.1*	---	Pseudogene
3q13	*RP11-10G15.4*	---	Pseudogene
3q13	*MTND5P16*	MT-ND5 pseudogene 16	LncRNA
3q21	*GS1-388B5.8*	---	Pseudogene
3q21	*RNA5SP58*	RNA, 5S Ribosomal Pseudogene 57	Pseudogene
3q22	*HSPA8P19*	Heat Shock 70kDa Protein 8 Pseudogene 19	Pseudogene
3q22	*PRR23B*	Proline Rich 23B	---
3q23	*ACTG1P1*	Actin Gamma 1 Pseudogene 1	Pseudogene
3q23	*RNU1-100P*	RNA, U1 Small Nuclear 100	Pseudogene
3q23	*RP11-68L1.2*	---	Antisense RNA
3q23	*CLSTN2-AS1*	CLSTN2 antisense RNA 1	Antisense RNA
3q25	*RP11-64D22.5*	---	LncRNA
3q25	*RP11-451G4.3*	---	LncRNA

3q25	*AADAC*	Arylacetamide deacetylase	Microsomal arylacetamide deacetylase
3q25	*PA2G4P4*	Proliferation-Associated 2G4 Pseudogene 4	Pseudogene
3q25	*RPL21P42*	ribosomal protein L21 pseudogene 42	Pseudogene
3q25	*PLCH1-AS1*	PLCH1 antisense RNA 1	Antisense RNA
3q25	*RP11-103G8.2*	ERICH6 antisense RNA 1	Antisense RNA
3q25	*IQCJ-SCHIP1-AS1*	IQCJ-SCHIP1 Readthrough Antisense RNA 1	Antisense RNA
3q26	*HMGN1P8*	high mobility group nucleosome binding domain 1 pseudogene 8	LncRNA
3q26	*RP11-152C17.1*	---	LncRNA
3q26	*RP11-298O21.2*	---	LncRNA
3q26	*RP11-298O21.6*	---	LncRNA
3q26	*RP11-816B4.1*	---	Pseudogene
3q26	*AC007620.3*	---	Antisense RNA
3q27	*RNU6-1105P*	RNA, U6 Small Nuclear 1105	Pseudogene
3q27	*RP11-573D15.2*	---	LncRNA
3q27	*DVL3*	Dishevelled, dsh homolog 3	Regulation of cell proliferation
3q27	*EIF2B5-AS1*	EIF2B5 antisense RNA 1	Antisense RNA
3q28	*GCNT1P3*	glucosaminyl transferase 1, core2 pseudogene 3	Pseudogene
3q29	*CPN2*	Carboxypeptidase N, Polypeptide 2	Carboxypeptidase
3q29	*SENP5*	SUMO1/sentrin specific peptidase 5	Post-translational modification of proteins
3q29	*MUC20*	Mucin 20, Cell Surface Associated	Glycoprotein of mucous barrier
3q29	*HES1*	Hairy enhancer of split 1	Transcription factor
3q29	*RP11-135A1.2*	---	Antisense RNA
4p13	*RN7SL691P*	RNA, 7SL, Cytoplasmic 691	Pseudogene
6p21	*RP5-973N23.4*	---	LncRNA
6p21	*CSNK2B/CK2B*	Casein kinase 2, beta subunit	Regulation of programmed cell death
6p21	*BTNL2*	Butyrophilin-Like 2	Negative regulation of T-cell proliferation
6p21	*CCHCR1*	Coiled-Coil Alpha-Helical Rod Protein 1	Regulation of keratinocyte proliferation
6p21	*HIST1H1T*	Histone Cluster 1, H1t	Epigenetic regulation of transcription
6p21	*HLA-DRA*	MHC complex, Class II, DR Alpha	Antigen presentation
6p22	*RP3-369A17.2*	---	Pseudogene
6p22	*VN1R11P*	Vomeronasal 1 Receptor 11 Pseudogene	Pseudogene
6p22	*MCCD1P1*	Mitochondrial coiled-coil domain 1 pseudogene 1	Pseudogene
6p22	*HIST1H2BC*	Histone Cluster 1, H2bc	Epigenetic regulation of transcription
6p22	*HIS1H2AK*	Histone Cluster 1, H2ak	Epigenetic regulation of transcription
6p22	*KAAG1*	Kidney Associated Antigen 1	---
6p24	*RP11-69L16.4*	---	Antisense RNA
6p25	*SERPINB1*	Serpin Peptidase Inhibitor, Class B , Mb1	Proteinase inhibition
6q12	*RP11-74E24.2*	Zinc finger CCCH-Type Domain-Containing-Like	Pseudogene
6q13	*KHDC1*	KH homology domain containing 1	Predicted membrane protein
6q21	*RP11-249L21.4*	---	Pseudogene
6q25	*AIRN*	Antisense of IGF2R Non-Protein Coding RNA	Regulation of transcription
6q25	*RP3-403L10.3*	---	Antisense RNA
7p22	*GPR146*	G Protein-Coupled Receptor 146	G protein coupled receptor
7p22	*AC073343.13*	---	Antisense RNA
7q11	*ABHD11*	Abhydrolase domain containing 11	---
7q11	*STX1A*	Syntaxin 1A	Docking of synaptic vesicles
7q21	*AC079781.7*	---	Pseudogene
11p15	*ART5*	ADP-Ribosyltransferase 5	ARG-specific ADP-ribosyltransferase
12p13	*PRH1*	Proline-Rich Protein HaeIII Subfamily 1	Inhibition of crystal calcium phosphates
12p13	*C1R*	Complement Component 1, R Subcomponent	Complement activation
12p13	*PTMS*	Parathymosin	Inhibition of linkage of H1 to chromatin
12q12	*RP11-313F23.3*	---	LncRNA
12q12	*CLUHP8*	clustered mitochondria homolog pseudogene 8	Pseudogene
12q14	*RAB11AP2*	RAB11A, member RAS oncogene family pseudogene 2	Pseudogene
12q22	*PGAM1P5*	Phosphoglycerate Mutase 1 Pseudogene 5	Pseudogene
12q23	*RNA5SP371*	RNA, 5S Ribosomal Pseudogene 371	Pseudogene
12q24	*RP11-968O1.5*	---	Sense overlapping
12q24	*RP11-428I12.1*	---	Pseudogene
12q24	*RP11-572C21.1*	---	LncRNA
12q24	*RP11-1028N23.3*	---	LncRNA
12q24	*RP11-121J20.1*	---	LncRNA
12q24	*DIABLO*	Diablo, IAP-Binding Mitochondrial Protein	Apoptosis promotion
12q24	*LRRC43*	Leucine Rich Repeat Containing 43	---
12q24	*RP11-64B16.4*	---	Antisense RNA
13q13	*EXOSC8*	Exosome Component 8	Transcriptional regulation
14q11	*AE000660.4*	---	Pseudogene
14q11	*OR4Q3*	Olfactory receptor, family 4, subfam. Q, mb 3	G-protein-coupled olfactory receptor
14q11	*TRAV-26-1*	T Cell Receptor Alpha Variable 26-1	TCR assembly
14q11	*TRAJ10*	T Cell Receptor Alpha Joining 10	TCR assembly

14q11	*TRAJ13*	T Cell Receptor Alpha Joining 13	TCR assembly
14q12	*RP11-468E2.6*	---	---
14q32	*HOMER2P1*	Homer Scaffolding Protein 2 Pseudogene 1	Pseudogene
14q32	*IGHVIII-2-1*	Immunoglobulin Heavy Variable (III)-2-1	BCR assembly
14q32	*IGHVIII-16-1*	Immunoglobulin Heavy Variable (III)-16-1	BCR assembly
14q32	*DCAF11*	DDB1 And CUL4 Associated Factor 11	Assembly of the DDB1–CUL4A ubiquitin ligase
15q15	*CATSPER2*	Cation Channel, Sperm Associated 2	Sperm motility and male fertility
15q25	*RP11-182J1.14*	---	Pseudogene
16p13	*NARFL*	Nuclear prelamin A recognition factor-like	Modulation of hypoxia-inducible factor-1*α* activity
16q22	*HAS3*	Hyaluronan Synthase 3	Synthesis of glycosaminoglycan hyaluronan
16q22	*CDH16*	Cadherin 16	Ca2+-dependent glycoprotein
16q22	*CLEC18B*	C-Type Lectin Domain Family 18, Member B	---
17p11	*LGALS9C*	Lectin, Galactoside-Binding, Soluble, 9C	Regulation of immune response
19p13	*LRG1*	Leucine-rich alpha-2-glycoprotein 1	Signal transduction, and cell adhesion
19p13	*ZNF414*	Zinc Finger Protein 414	Transcriptional regulation
19p13	*AC008686.1*	---	Uncharacterized protein
22q12	*RP5-1170K4.7*	---	Antisense RNA
22q13	*RN7SKP252*	---	misc RNA
22q13	*RPL35P8*	Ribosomal Protein L35 Pseudogene 8	Pseudogene
22q13	*OGFRP1*	opioid growth factor receptor pseudogene 1	LncRNA
22q13	*ADSL*	Adenylosuccinate lyase	*De novo* purine biosynthesis

Supplemental figures

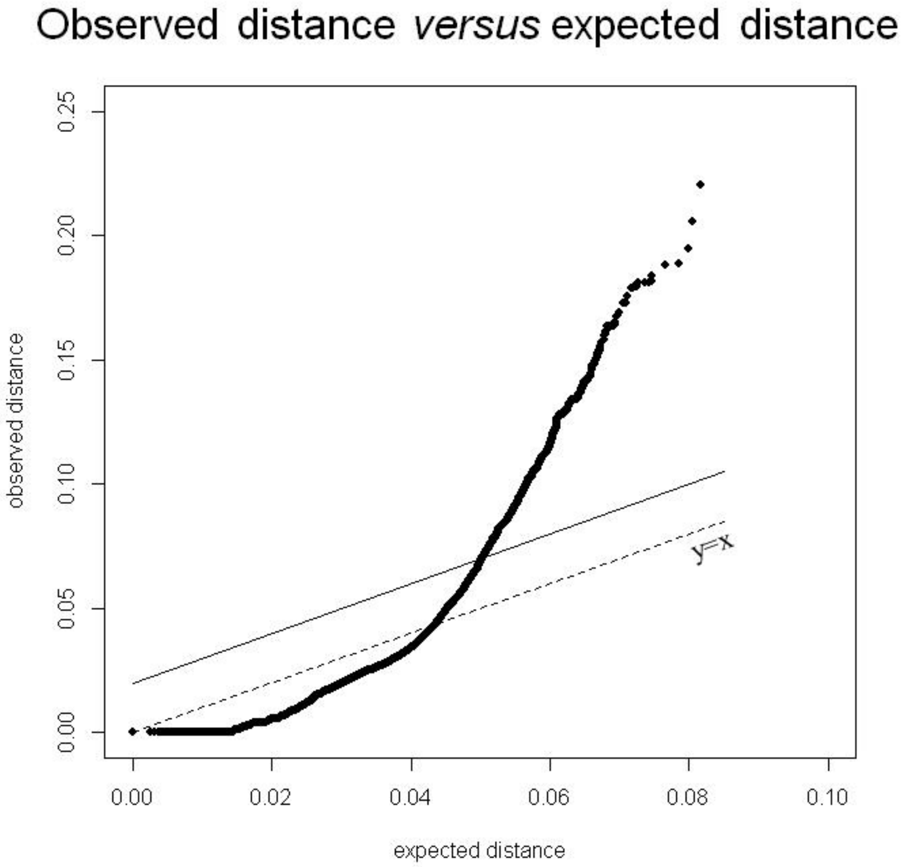

Supplemental Figure 1: observed distance versus expected distance using the permutation-based SAM method. One hundred and forty-eight genes presented with an observed distance superior to the expected distance, with a false discovery rate < 0.05.
